# Equity in HIV mental health research: a call to action

**DOI:** 10.1038/s41380-022-01748-8

**Published:** 2022-09-02

**Authors:** Arish Mudra Rakshasa-Loots

**Affiliations:** 1 Edinburgh Neuroscience, School of Biomedical Sciences, College of Medicine and Veterinary Medicine, The University of Edinburgh, Edinburgh, UK; 2 Family Centre for Research with Ubuntu (FAMCRU), Department of Paediatrics and Child Health, Stellenbosch University, Cape Town, South Africa

**Keywords:** Neuroscience, Psychiatric disorders

## Abstract

The brain remains a key reservoir of latent HIV infection, and people living with HIV (PLWH) face a high risk for cognitive impairment and psychiatric disorders. Although the burden of HIV infection and co-morbidities is greatest in the Global South, a large proportion of HIV mental health research is carried out in the Global North. Large, well-funded observational cohort studies exploring HIV-associated psychopathology generally involve participant groups from WEIRD (Western, educated, industrialised, rich and democratic) settings. The socioeconomic status and institutional access afforded to these participant groups on average does not reflect those of the majority of beneficiaries of HIV mental health research. This misalignment may lead to limitations in generalising findings and developing effective interventions to improve the mental health of PLWH. Here, I offer recommendations to actively cultivate authentic diversity and inclusion in the field, with four focus points: (1) for funding bodies, to actively invest in neuroscientists in the Global South for investigations of HIV-related psychopathology; (2) for scientific publishers, to fund professional support services for researchers in the Global South; (3) for academic institutions, to facilitate meaningful, equitable collaborations with researchers in the Global South and incentivise studies with diverse participant groups; and (4) for individual neuroscientists, to actively cite and converse with colleagues in the Global South, tackle personal biases in those conversations, and avoid overgeneralising findings from primarily WEIRD participant groups.

## Introduction

The mental health of people living with HIV (PLWH) represents an important yet challenging frontier in neurovirology. Advancements in immunology and drug development have made viral suppression possible for PLWH who have access to combination antiretroviral therapy (cART). However, the brain remains a key reservoir of latent HIV infection even in virally-suppressed patients, and HIV-related neurotoxicity leads to substantial neuronal damage in PLWH [1]. Chronic HIV infection leads to a cluster of symptoms associated with neurocognitive impairment, notably memory issues and slowness, which have been collectively termed HIV-Associated Neurocognitive Disorders [2]. PLWH also face a remarkably high risk of depression, with studies estimating the prevalence of HIV-associated depression to be over 50% [3]. Together, these neuropathologies significantly reduce the quality of life for a large proportion of PLWH.

## Inequity in HIV mental health research

An estimated 5.8% of PLWH are in western/central Europe or North America, whereas 67.7% of PLWH are in Africa or the Middle East [4]. Despite this, a review of 68,808 studies found that over 80% of research into HIV published between 2010–2017 was produced in North America and Europe [5]. Authorship of HIV research is clustered in high-income countries in North America and Europe [6], whereas the greatest burden of HIV infection lies in the Global South, where access to latest-generation cART and psychological healthcare is also limited. Similarly, although the global proportion of PLWH who are women is estimated to be 53% [4], only 19.17% of participants in studies of mental health issues amongst PLWH are women [7]. This divide represents a major gap in the field: studies of HIV-associated psychopathology continue to be carried out in settings that do not accurately capture the complex interrelationships between health, society, and culture that drive HIV mental health co-morbidities.

**Fig. 1:**
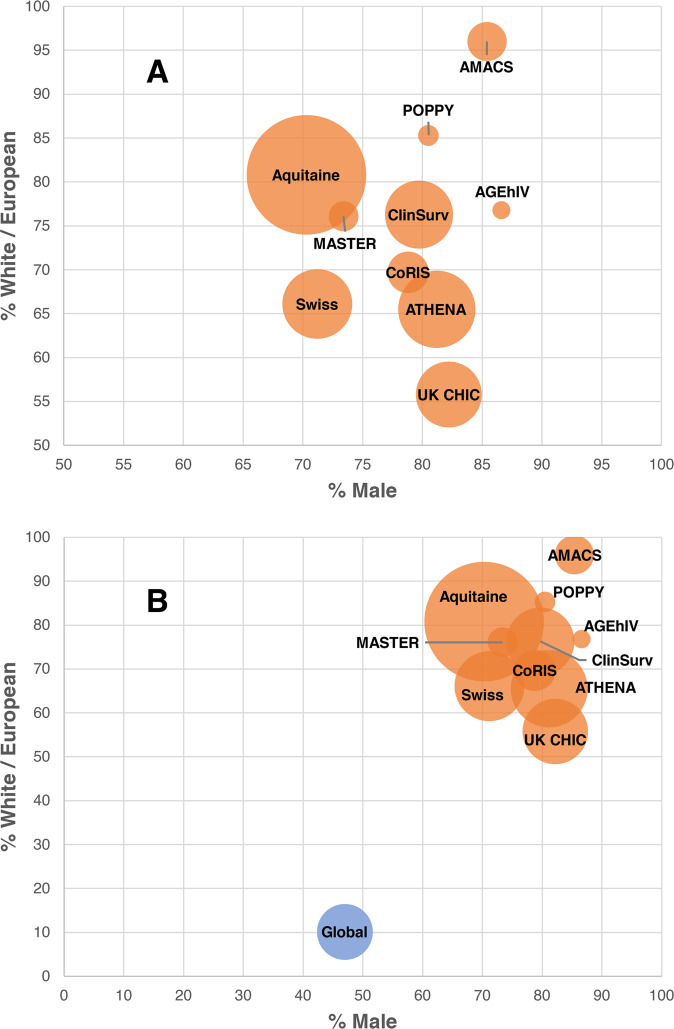
Sex and ethnicity distribution in samples of ten European HIV cohort studies with *N* > 1000 each. **A** For cohort studies alone, and **B** alongside global estimates of % PLWH who are White and male. Size of each bubble is proportional to the total sample size of each study. For reference, global estimates of % White and % male PLWH using UNAIDS data are also shown. Data used to produce this visualisation, along with references for each cohort study, may be found in Supplementary Table 1.

Not all clinical studies are created equal—and in HIV neurovirology, especially, studies often involve participant groups from Western, educated, industrialised, rich, and democratic (WEIRD) backgrounds. The socioeconomic status and institutional access afforded to many of these participant groups in large, well-funded observational cohort studies do not reflect those of the majority of people who are affected by HIV-associated psychopathologies and would thus be the beneficiaries of this research. Figure 1 highlights the distribution of sex and ethnicity in ten large observational cohort studies of HIV in Europe. (These studies were selected as examples since they each included more than 1000 participants and assessed HIV co-morbidities. Many of these cohorts included specific measurements of neuropsychiatric co-morbidities.) The total sample size for these ten cohorts was *N* = 125,107. Within this total sample, a remarkable 78.9% of participants were male, and 74.8% were White/European.

It may be argued that this skew in participant profiles may be because these studies are set in countries with predominantly White populations. That would not, however, explain why such a substantial proportion of study participants are male. This is particularly concerning since gender is a significant factor predicting psychiatric morbidity amongst PLWH, such that women living with HIV are more likely to experience severe depression than men with HIV [8, 9].

The impact of this lack of gender and ethnic diversity cannot be understated. Follow-up studies rely on data obtained from these large cohorts to investigate new questions, and ultimately reproduce the same limitations in participant demographic distribution as the original studies. For instance, the AGE_*h*_IV (Netherlands) and Pharmacokinetic and clinical Observations in PeoPle over fiftY (POPPY, UK) cohorts were established to explore HIV co-morbidities. Both cohorts included assessments of mental health and neuropsychiatric outcomes. The COmorBidity in Relation to AIDS (COBRA) study utilised data from these parent studies to further explore age-associated co-morbidities with HIV such as cognitive impairment. Reflecting the lack of diversity in participant groups within the parent studies, the participant sample included in the COBRA cohort was 92.7% White and 92.8% male [10]. Large cohort studies with non-diverse participant groups may thus encourage citation of, and secondary analyses based on, data from homogeneous samples with limited generalisability.

Crucially, findings from these studies may be used to inform policy worldwide, especially as studies with sample sizes in the thousands may be perceived as inherently more reliable. Together with the lack of investment in HIV cohort studies in the Global South, this means that any evidence-based public health policies related to HIV, even in countries where most PLWH are not White or male, must rely on findings from these European cohort studies. Key sociocultural factors such as access to latest generation cART, degree of social stigma associated with HIV, and lifestyle influence progression of neuropsychiatric co-morbidities and differ considerably across the globe. Studies in the Global North leverage participant groups with a narrow sociocultural profile, which limits the application and relevance of these findings to policymaking in the Global South.

Moreover, prevalence rates of psychiatric co-morbidities can vary drastically by sex, ethnicity, income, and geography [11], with studies in WEIRD settings often reporting lower rates of mental health issues. Without rigorous comparative research in diverse world regions and conscious investment in improving the sex distribution in participant samples, findings from primarily male samples and WEIRD settings may be the only evidence available to inform public health policy. Given varying prevalence rates, this evidence may therefore drive public sector support away from critical psychosocial services for PLWH, and women with HIV specifically, in regions where prevalence rates—and thus need for support and public health investment—may in fact be much higher.

Finally, a lack of representation and local capacity-building may encourage “helicopter” or neo-colonial research, which can open the door for unethical or potentially harmful clinical trials being carried out with populations in the Global South. This is not unprecedented [12]. Improving representation within the field would allow for the involvement of researchers with cultural or historical understanding, thus facilitating better monitoring of global trials and ensuring equitable study design. Similarly, improving diversity of participant groups has the potential to enhance our understanding of mental health disparities, better track psychiatric co-morbidities that disproportionately affect certain groups of PLWH, and more equitably disseminate findings from research [13]. Therefore, there is much to be gained from addressing the lack of diversity within studies of HIV mental health.

## Where do we go from here?

Given that much of HIV neurovirology research is carried out in the Global North, and since participant groups in many of these large HIV cohort studies are overwhelmingly White and male, there is an urgent need to pursue authentic diversity and inclusion in the field. As a growing number of institutions across the world have committed to enhancing diversity and inclusion in science, following are some recommendations to actualise these commitments in the context of HIV and mental health research.

**Funding bodies** must actively invest in scientists in the Global South to investigate HIV-related psychopathology. This is increasingly urgent given recent UK funding cuts to foreign research that have severely impacted researchers in the Global South [14]. Setting up HIV cohort studies, particularly those with large (>1000) sample sizes, is an expensive affair anywhere, and doing so is especially challenging in formerly-colonised nations where resources have been drained over several generations. Being at the favourable end of currency exchange rates, funding bodies in the Global North are well-positioned to create significant impact with relatively small investments. Therefore, these agencies must prioritise funding for research and administrative costs associated with establishing longitudinal HIV cohorts and developing language- and culture-specific research tools in the Global South.

An initiative funded by the National Institutes of Health placed US scientists from minority ethnic backgrounds into resource-limited settings through the HIV Vaccine Trials Network at domestic and international placement sites [15]. The rationale behind this project was that scientists from minority ethnic backgrounds may better understand the issues affecting underserved communities and be able to build relationships with community members. This initiative is an example of successful investment in HIV research that generated tangible professional developments for the scientists who completed these placements.

However, going forward, the training and professional development of scientists from local communities must be prioritised. It is important to avoid homogenising individuals across artificial categories of race or ethnicity in such cross-border initiatives. For instance, it is unfair to assume that a Black scientist from the US can automatically relate to and engage with Black research participants in a placement in South Africa. Instead, investing in empowering researchers from indigenous backgrounds to conduct and disseminate research in their communities is necessary to truly build capacity in the Global South and move away from a “top-down” power dynamic.

Funding decisions can be shaped by the ability to navigate complex and often unwritten rules of grant writing, which are inaccessible to researchers from minority backgrounds without significant mentorship or generational links. Investing in local capacity-building and mentorship will allow researchers from the Global South to gain access to these funding mechanisms and help create a more equitable funding landscape. In turn, this will empower these researchers to take the lead in driving the research agenda.

Funding agencies are well-situated to foster a culture of substantive self-critique. It cannot simply be enough to say that 90% of participants in a study were White or male; authors must justify their participant profiles or offer comparisons with local or regional demographics. In doing so, researchers in the Global North can avoid overgeneralising findings from primarily WEIRD participant groups or overlooking consistent and systematic barriers that may prevent certain groups from participating in research. Instead, researchers can be empowered to embrace honest critiques of their own work when discussing their findings in publications. However, this is only possible when funding bodies encourage such reflections and commit to not penalising researchers for these self-critiques in future applications. This would especially allow early career researchers who may wish to seek funding to establish more diverse HIV cohorts in future to still critique their existing work honestly without fear of losing funding.

**Scientific publishers** must increase financial and professional support for researchers from the Global South. Currently, article processing fees charged to publish in open access journals represent a substantial hurdle for authors from the Global South. While some publishers offer open access waivers, researchers from the Global South are often ineligible for these if they take lead on projects which involve collaborators in the Global North. Publishers must continue to expand the open access waiver programmes for researchers from low- and middle-income countries in ways that allow for cross-border collaborations.

Another relatively straightforward way in which publishers can demonstrate their commitment to promoting diversity and inclusion in research is by funding English language editing for scientists in the Global South. Researchers who are non-native English speakers face a dual challenge: having to pay for English language editing services adds expenses to already stretched research budgets, but not paying for such services (or relying on unpaid labour of colleagues) runs the risk of robust, well-designed research being dismissed or perceived as “less than” amongst peers in the Global North due to superficial grammatical flaws. Providing free access to English language editing services before, during, and after the manuscript review process is thus a concrete way in which publishers can increase their support of researchers in the Global South. In turn, publishers who use a small proportion of their profits to offer funding for English language editing will gain a competitive edge over others, as authors will view such material support favourably and will be more likely to submit to, and publish with, publishers who offer these services.

**Academic institutions**, particularly those in the Global North, must actively facilitate collaborations with researchers in the Global South and incentivise studies with diverse participant groups. Fostering collaborations with colleagues in different countries takes substantial work for scientists. Research institutions can offer meaningful support in turn by simplifying the process of establishing contracts to achieve these collaborations and giving researchers accessible resources on the relevant legal and administrative requirements.

Institutions can additionally promote capacity-building through strategic collaborations that create opportunities for researchers in the Global South to access high-quality professional development opportunities, training, and specialised equipment. Development and execution of studies involving diverse participant samples may also be prioritised in key academic processes such as tenure or institutional funding support. Improving diversity and inclusion in participant samples should not become an obligation or a chore that scientists in the Global North dread; that would be deeply counter-productive. Rather, funding bodies and academic institutions are uniquely well-placed to incentivise this pursuit and cultivate a positive ideal towards which researchers may strive.

Finally, **individual scientists** must actively cite and converse with colleagues in the Global South. Robust, reproducible research using gender- and ethnically-diverse participant samples must be highlighted at HIV and neurovirology conferences in the Global North, so that new collaborations and follow-up studies may emerge from this work. It is not sufficient to merely interact with researchers in the Global South; researchers must actively develop respectful and culturally-sensitive communication skills so as to tackle their own personal biases in these collaborations. Perhaps most crucially, individual scientists must listen to colleagues and, more broadly, people with lived experience of HIV and psychiatric co-morbidities from the Global South. By taking the lead from voices in the Global South, researchers in the Global North can participate in research that is inclusive and impactful.

When interviewing for a highly prestigious immunology PhD programme in the US a few years ago, I explained to one of the faculty that I planned to study HIV, who responded: “Oh, so you’re gay?” That response is less unusual than one would hope, and it reflects a common assumption that HIV research is only important to the LGBTQ+ community or to people in sub-Saharan Africa. We cannot allow these stereotypes to persist in the field. As we work to establish large cohort studies with more diverse participant groups, it is important for individual researchers to challenge these assumptions and be respectful in their collaborations with colleagues in the Global South.

## Conclusion

Sociocultural aspects play an important role in HIV-associated psychopathologies, and there is compelling evidence that socioeconomic factors interact significantly with biology to predict disease progression [16]. Therefore, it is critical to replicate findings in diverse populations. Improving mental healthcare available to PLWH requires concerted, global interventions, which must necessarily be founded on robust and inclusive research. Diversifying participation in research in this field will require commitment from stakeholders across sectors. Furthermore, research on HIV mental health co-morbidities must include and empower the voices of PLWH themselves to shape the research agenda. The pursuit of authentic diversity and inclusion in HIV mental health research will enhance the reproducibility and generalisability of findings and ultimately benefit researchers, funders, and public health alike.

## Supplementary information

Supplementary Table 1

## Data Availability

Study sample characteristics associated with Fig. 1 may be found in Supplementary Table 1. No other data were generated for this article.
