# Supplementary material for: Equity in HIV mental health research: a call to action
Source: Mol Psychiatry. 2022 Sep 2;27(12):4824–8. doi: 10.1038/s41380-022-01748-8 (PMC9763108; doi:10.1038/s41380-022-01748-8)
Supplement: Supplementary file 1 — Supplementary Table 1 [file 41380_2022_1748_MOESM1_ESM.docx]

**Supplementary Table 1: Sex and ethnicity distribution in samples of ten European HIV cohort studies.**

| **Study** | **Region** | **Total Participants** | **% Male** | **% White/European** | **Reference** |
| --- | --- | --- | --- | --- | --- |
| AGE_h_IV | Netherlands | 1064 | 86.6 | 76.8 | Schouten et al., 2014 |
| AMACS^[[1]](#footnote-1)^ | Greece | 4910 | 85.4 | 96.0 | Pantazis et al., 2018 |
| Aquitaine^[[2]](#footnote-2)^ | France | 45 999 | 70.3 | 80.75^[[3]](#footnote-3)^ | Mary-Krause et al., 2014 |
| ATHENA | Netherlands | 19 035 | 81.2 | 65.5 | Boender et al., 2018 |
| ClinSurv | Germany | 14 874 | 79.7 | 76.3^[[4]](#footnote-4)^ | Bätzing-Feigenbaum et al., 2011 |
| CoRIS | Spain | 5514 | 78.8 | 69.7^[[5]](#footnote-5)^ | Sobrino-Vegas et al., 2011 |
| MASTER^[[6]](#footnote-6)^ | Italy | 2877 | 73.4 | 76.1^[[7]](#footnote-7)^ | Torti et al., 2017 |
| POPPY | UK, Ireland | 1377 | 80.5 | 85.3 | Bagkeris et al., 2018 |
| Swiss | Switzerland | 15 624 | 71.2 | 66.1 | Schoeni-Affolter et al., 2010 |
| UK CHIC | UK | 13 833 | 82.2 | 55.8 | UK Collaborative HIV Cohort Steering Committee, 2004 |
| SUM |  | 125 107 |  |  |  |
| MEAN |  |  | 78.9 | 74.8 |  |
| UNAIDS Estimates | Global |  | 47 | 10.1^[[8]](#footnote-8)^ | UNAIDS |

**References**

Bagkeris E, Burgess L, Mallon PW, Post FA, Boffito M, Sachikonye M, et al. Cohort profile: The Pharmacokinetic and clinical Observations in PeoPle over fiftY (POPPY) study. International Journal of Epidemiology. 2018;47(5):1391-2e.

Bätzing‐Feigenbaum J, Kollan C, Kühne A, Matysiak‐Klose D, Gunsenheimer‐Bartmeyer B, Hamouda O, et al. Cohort profile: the German ClinSurv HIV project–a multicentre open clinical cohort study supplementing national HIV surveillance. HIV medicine. 2011;12(5):269-78.

Boender TS, Smit C, van Sighem A, Bezemer D, Ester CJ, Zaheri S, et al. AIDS Therapy Evaluation in the Netherlands (ATHENA) national observational HIV cohort: cohort profile. BMJ open. 2018;8(9):e022516.

Mary-Krause M, Grabar S, Lievre L, Abgrall S, Billaud E, Boué F, et al. Cohort profile: French hospital database on HIV (FHDH-ANRS CO4). International journal of epidemiology. 2014;43(5):1425-36.

Pantazis N, Chini M, Antoniadou A, Sambatakou H, Skoutelis A, Gargalianos P, et al. The HIV patient profile in 2013 and 2003: Results from the Greek AMACS cohort. Plos one. 2018;13(9):e0203601.

Schoeni-Affolter F, Ledergerber B, Rickenbach M, Rudin C, Günthard HF, Telenti, A, et al. Cohort profile: the Swiss HIV Cohort study. International journal of epidemiology. 2010;39(5):1179-89.

Schouten J, Wit FW, Stolte IG, Kootstra NA, van der Valk M, Geerlings SE, et al. Cross-sectional comparison of the prevalence of age-associated comorbidities and their risk factors between HIV-infected and uninfected individuals: the AGEhIV cohort study. Clinical Infectious Diseases. 2014;59(12):1787-97.

Sobrino-Vegas P, Gutierrez F, Berenguer J, Labarga P, Garcia F, Alejos-Ferreras B, et al. The Cohort of the Spanish HIV Research Network (CoRIS) and its associated biobank; organizational issues, main findings and losses to follow-up. Enfermedades infecciosas y microbiologia clinica. 2011;29(9):645-53.

Torti C, Raffetti E, Donato F, Castelli F, Maggiolo F, Angarano G, et al. Cohort Profile: Standardized management of antiretroviral therapy cohort (MASTER Cohort). International Journal of Epidemiology. 2017;46(2):e12-e.

UK Collaborative HIV Cohort Study Committee. The creation of a large UK‐based multicentre cohort of HIV‐infected individuals: the UK Collaborative HIV Cohort (UK CHIC) Study. HIV medicine. 2004;5(2):115-24.

UNAIDS. Fact sheet - Latest global and regional statistics on the status of the AIDS epidemic.; 2021.

1. Data from the 2013 study wave. [↑](#footnote-ref-1)
2. Data from the 2001 ANRS-CO4 study wave. [↑](#footnote-ref-2)
3. Race/ethnicity not explicitly reported; 80.75% of enrolled participants were reported as having country of origin “France” but may not necessarily have been White. [↑](#footnote-ref-3)
4. Race/ethnicity not explicitly reported; 76.3% of enrolled participants were reported as “from Germany” but may not necessarily have been White. In comparison, 10.3% of participants were “from sub-Saharan Africa”. [↑](#footnote-ref-4)
5. Race/ethnicity not explicitly reported; 69.7% of enrolled participants were reported as “Spanish” but may not necessarily have been White. [↑](#footnote-ref-5)
6. Data from 2010-13 study wave. [↑](#footnote-ref-6)
7. Race/ethnicity not explicitly reported; 76.1% of enrolled participants were reported as having country of origin “Italy” but may not necessarily have been White. [↑](#footnote-ref-7)
8. Race/ethnicity not explicitly reported. 10.1% of people living with HIV in UNAIDS reports are in western, central, and eastern Europe, central Asia, and North America. Not all PLWH in these regions may be White, and some PLWH in other regions may be White. [↑](#footnote-ref-8)
